# Fluorescence-Guided Resection of Malignant Glioma with 5-ALA

**DOI:** 10.1155/2016/6135293

**Published:** 2016-06-27

**Authors:** Sadahiro Kaneko, Sadao Kaneko

**Affiliations:** ^1^Department of Neurosurgery, Tomakomai City Hospital, No. 5-20, 1-chome, Shimizutyou, Tomakomai, Hokkaido 053-0034, Japan; ^2^Department of Neurosurgery, Kashiwaba Neurosurgical Hospital, No. 7-20, 15-chome, 1-jyou, Tsukisamuhigashi, Toyohira-ku, Sapporo, Hokkaido 062-5813, Japan

## Abstract

Malignant gliomas are extremely difficult to treat with no specific curative treatment. On the other hand, photodynamic medicine represents a promising technique for neurosurgeons in the treatment of malignant glioma. The resection rate of malignant glioma has increased from 40% to 80% owing to 5-aminolevulinic acid-photodynamic diagnosis (ALA-PDD). Furthermore, ALA is very useful because it has no serious complications. Based on previous research, it is apparent that protoporphyrin IX (PpIX) accumulates abundantly in malignant glioma tissues after ALA administration. Moreover, it is evident that the mechanism underlying PpIX accumulation in malignant glioma tissues involves an abnormality in porphyrin-heme metabolism, specifically decreased ferrochelatase enzyme activity. During resection surgery, the macroscopic fluorescence of PpIX to the naked eye is more sensitive than magnetic resonance imaging, and the alert real time spectrum of PpIX is the most sensitive method. In the future, chemotherapy with new anticancer agents, immunotherapy, and new methods of radiotherapy and gene therapy will be developed; however, ALA will play a key role in malignant glioma treatment before the development of these new treatments. In this paper, we provide an overview and present the results of our clinical research on ALA-PDD.

## 1. Introduction

Malignant gliomas consist of anaplastic astrocytoma (WHO grade III) and glioblastoma (WHO grade IV) and possess a lethal prognosis. Despite surgery, radiotherapy, and chemotherapy, the median survival time for a patient with glioblastoma is only 15.0 months, and it is not much better for patients with anaplastic astrocytoma [1].

Malignant gliomas are very difficult to treat. There is clearly a need for new, effective, and safe treatments for malignant glioma.

The remote metastasis of malignant gliomas to extracranial organs is rare, and therapeutic efficacy depends on local control of the malignant glioma in the brain.

On the other hand, the resection rate of glioma is believed to affect patient prognosis. The Committee of Brain Tumor Registry of Japan reported on the relationship between the resection rate of malignant glioma and patient survival time. After total resection of the tumor, the 5-year survival rate was 20.4%; however, after resecting 50% of the tumor, the 5-year survival rate was 3.8% [1]. Total resection of the tumor means that the enhancing tumor shadow completely disappears on magnetic resonance imaging (MRI) after surgery.

On MRI, malignant gliomas are well-circumscribed tumors with regional necrosis surrounded by viable invading tissues and marginal contrast enhancement. However, it is well known that infiltrating glioma cells exist in the brain adjacent to the tumor, 2-3 cm away from the main body of the glioma, and show contrast enhancement on MRI. Moreover, 80%–90% of glioblastoma recurrences occur from the brain adjacent to the tumor. MRI can fail to detect these infiltrating glioma cells in the brain adjacent to the tumor. In these respects, malignant gliomas have features that are in striking contrast to other malignant tumors elsewhere in the body.

Extensive resection of malignant glioma, including normal tissue, has to be limited to prevent the possibility of brain dysfunction after surgery. This is especially true for tumors located in the eloquent brain areas of the speech or motor functional centers of the brain in which serious complications, such as paralysis or speech disturbance, can occur after surgery. Due to the difficulty of extensive resection of malignant glioma, residual malignant glioma tissue is often observed despite tumor resection.

Lacroix et al. [2] reported on the relationship between the extent of the resection and the survival time in 416 patients with glioblastoma. A significant survival advantage was associated with resection of >98% of the tumor volume (median survival: 13 months) compared with resection of <98% of the tumor volume (median survival: 8.8 months, *p* < 0.0001). In addition, Keles et al. [3] reported on the relationship between the volume of the residual tumor tissue and the vital prognosis after removing the brain tumor in 107 glioblastoma patients. It was found that the median survival time for patients with complete disappearance of the tumor shadow, as assessed by CT or MRI, was 93 weeks. The median survival time in patients with 10, 10–20, and >20 cc of residual tumor volume was 68.7, 49.0, and 50.8 weeks (*p* < 0.0001), respectively. Furthermore, they observed that the volume of the residual tumor greatly influenced the time-to-recurrence of the tumor. These results indicate that increasing the resection rate of gliomas prolongs the patient survival time. Therefore, to ensure the best prognosis after surgical removal in patients with malignant gliomas, neurosurgeons should aim to remove >98% of the tumor and at least ensure that the residual tumor volume is <10 cc while, at the same time, preserving the function of the surrounding normal brain. The main aim of surgery is total removal of the glioma, which is the contrast-enhancing area on MRI. Unfortunately, this goal is achieved in <35% of cases. Therefore, it is visually very difficult to differentiate glioma tissue from normal tissue based on its color or hardness [4].

**Figure 1:**
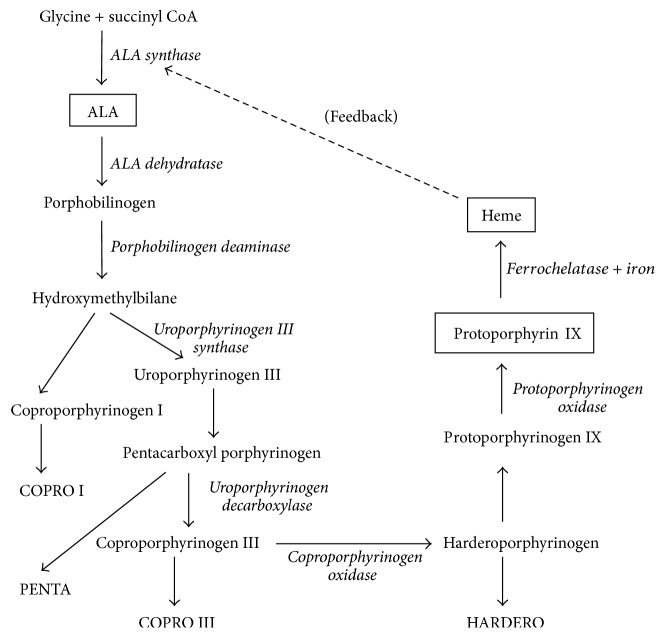
The simplified synthetic pathway of ALA-induced PpIX. ALA is an endogenous substance. It is incorporated into the mitochondria, repeatedly transported between the cytoplasm and the mitochondria, and finally used for heme synthesis. ALA is converted into porphobilinogen by the enzyme ALA dehydratase, and porphobilinogen is sequentially converted into hydroxymethylbilane, uroporphyrinogen III, coproporphyrinogen III, protoporphyrinogen IX, and finally PpIX by the enzyme protoporphyrinogen oxidase. The final product heme is synthesized from PpIX and iron (Fe) by the enzyme ferrochelatase (FeC). This biosynthetic pathway is considered to be the same in all organisms (PENTA: pentacarboxyl porphyrin, COPRO I or III: coproporphyrin I or III, and HARDERO: harderoporphyrin).

5-Aminolevulinic acid (ALA) appears to be a new and promising material in the field of malignant glioma treatment. Because ALA is specifically taken up by malignant glioma cells and used for the biosynthesis of protoporphyrin IX (PpIX) (Figure 1), there is an abundant and specific accumulation of PpIX in malignant glioma cells. The tumor fluorescence by excitation of violet-blue light is useful for photodynamic diagnosis (PDD) of the glioma at the time of surgery. ALA-PDD can distinguish the infiltrating area, which is very difficult to distinguish using gadolinium-enhanced MRI (Gd-MRI), and the following treatment is now possible. An exclusive removal of the glioma without functional damage to the brain is possible by intraoperative PDD using ALA through distinguishing malignant glioma tissue from normal brain tissue with the naked eye during surgery. This technique is known as fluorescence-guided resection of the glioma [5].

## 2. Materials and Methods

### 2.1. Patients

A total of 217 biopsy specimens in 125 malignant glioma patients were obtained in both fluorescent and nonfluorescent tissue. To clarify the relationship of the malignancy and the amount of PpIX, we obtained WHO grade of each glioma tissue, which was confirmed by pathologist. All patients provided written informed consent, and the study was approved by the ethics committees of our hospital.

### 2.2. High Performance Liquid Chromatography

The amount of >25 types of intracellular trace elements and iron (Fe^++^) was measured using Kondo's method [6].

### 2.3. Pathological Examination

Histopathological examination was performed using hematoxylin and eosin (HE) staining. Cellular proliferation was assessed using the MIB-1 index by immunohistochemistry.

### 2.4. Statistical Analysis

The *χ* ^2^ test was used to compare the distribution of the categorical variables. In all analyses, a *p* value of <0.05 (two-sided) was considered to be statistically significant. All experiments were conducted more than three times with similar results.

### 2.5. Treatment

#### 2.5.1. Dosage and Timing of ALA Administration

20 mg/kg ALA was dissolved in 50 mL of 5% glucose solution and orally administered to the patient, approximately 1 h prior to the induction of anesthesia. The chemical formula of 5-ALA (5-aminolevulinic acid hydrochloride, Cosmo Bio Co., Ltd., Tokyo, Japan) is C_2_H_9_NO_3_–HCl, and its molecular weight is 167.59.

#### 2.5.2. Light Protection (Photosensitization of the Skin)

It is well known that photosensitization of the skin lasts approximately 24 h after ALA administration. In our practice of using ALA, direct exposure of the patient to sunlight or especially strong room light must be avoided; however, no special care is needed for the patient in general indoor illumination. However, during or after surgery, when an oximeter using LED is attached to the patient, care must be taken to avoid burns from part of the LED light touching the skin.

#### 2.5.3. Light Illumination

Violet-blue light was used as the excitation light because it irradiates at the wavelength of the Soret band at which PpIX shows very strong absorbance. The surgical field was irradiated with violet-blue light from a xenon lamp (Superlux 301, Carl Zeiss Japan, Tokyo, Japan) through an optical fiber that was equipped with a filter (Nikon EX405/10, 400–410 nm, Nikon Co., Tokyo, Japan) at the distal tip. It is possible to use other excitation light sources such as VLD-M1 (405 nm Diode Laser System, M&M Co., Tokyo, Japan) and surgical microscopes (OPMI Pentero, Carl Zeiss Japan) equipped with an excitation light source apparatus.

#### 2.5.4. Observation of the Fluorescence Intensity and Spectrum during Surgery

The three factors that influence fluorescence observation are distance, angle, and shading. All of these factors should be kept in mind when performing photoillumination (Figure 4(a)). Fluorescence, which is difficult to observe macroscopically, can be confirmed by simultaneously measuring the fluorescence spectrum. The presence or absence of fluorescence is determined by analyzing the fluorescence spectrum R/G ratio. The R/G ratio shows the ratio of the amplitude of the red PpIX fluorescence from the glioma tissues and the green autofluorescence from the glioma tissues in a spectrum (Figure 4(b)).

## 3. Results

### 3.1. Porphyrin Metabolism and PpIX Content in Malignant Glioma Cells

#### 3.1.1. Histological Examination

For the analysis of the intracellular trace elements, a total of 42 tissue samples were used in the biopsy specimens. In 28 of the 42 tissue samples, glioblastoma was histologically confirmed and fluorescence was observed. In 14 of the 42 tissue samples, no tumor cells and no fluorescence were observed, and they were used as controls in the following results.

#### 3.1.2. Is the Substance Emitting the Fluorescence PpIX?

**Figure 2:**
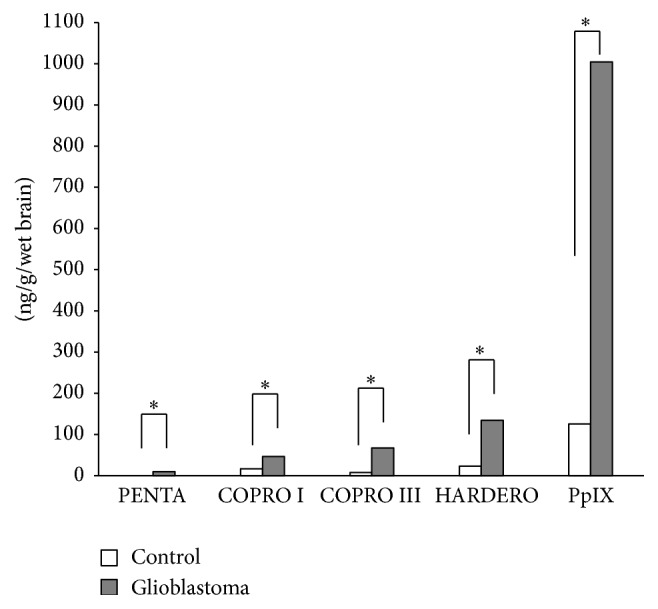
The amount of porphyrins in the control brain tissue and glioblastoma tissue. The white bar shows control tissue from a brain tumor patient (*n* = 14), and the gray bar shows glioblastoma (*n* = 28) (^*∗*^ *p* < 0.05) (PENTA: pentacarboxyl porphyrin, COPRO I or III: coproporphyrin I or III, HARDERO: harderoporphyrin, and PpIX: protoporphyrin IX).

In the human glioblastoma tissue and control brain tissue samples, the amounts of each porphyrin and the relevant enzyme activities of porphyrin metabolism were measured. The amount of all porphyrins was increased in the glioblastomas that emitted strong fluorescence after ALA administration compared with control brain tissue without fluorescence. On average, the amounts of coproporphyrin and harderoporphyrin increased by approximately twofold to ninefold and that of PpIX by sixfold to ninefold. PpIX was in the greatest quantity for these porphyrins at more than 10 times (Figure 2). Therefore, we can definitely say that the substance emitting the fluorescence is PpIX. Harada et al. [7] reported that PpIX shows a very strong absorbance band at a wavelength of approximately 405 nm wave (Soret band) and four absorbance bands between the wavelengths of 480 and 650 nm (Q bands). That is, if tissues containing PpIX are excited using violet-blue light at a wavelength of approximately 405 nm, a strong red fluorescence band at a wavelength of 635 nm is observed in the tissue. Although normal tissues also synthesize PpIX, fluorescence is not observed macroscopically in these tissues because the amount of PpIX is very small. After ALA administration, PpIX synthesis is observed; however, PpIX accumulation is not observed in normal brain tissue. In malignant glioma tissues, PpIX synthesis and accumulation are observed. This means that, based on the amount of accumulated PpIX, differentiation of malignant tumor tissue from normal brain tissue is possible. PpIX emits red fluorescence after excitation with violet-blue light, and this fluorescence can be macroscopically recognized. Therefore, tissues with red fluorescence that contain large amounts of PpIX are very likely to be tumor tissue, while those without red fluorescence that contain little PpIX are probably normal tissue. Many studies have addressed the question as to why a large amount of the ALA metabolite PpIX is specifically incorporated and accumulated in malignant glioma cells.

#### 3.1.3. Why Does PpIX Excessively Accumulate in Malignant Glioma Cells?

**Figure 3:**
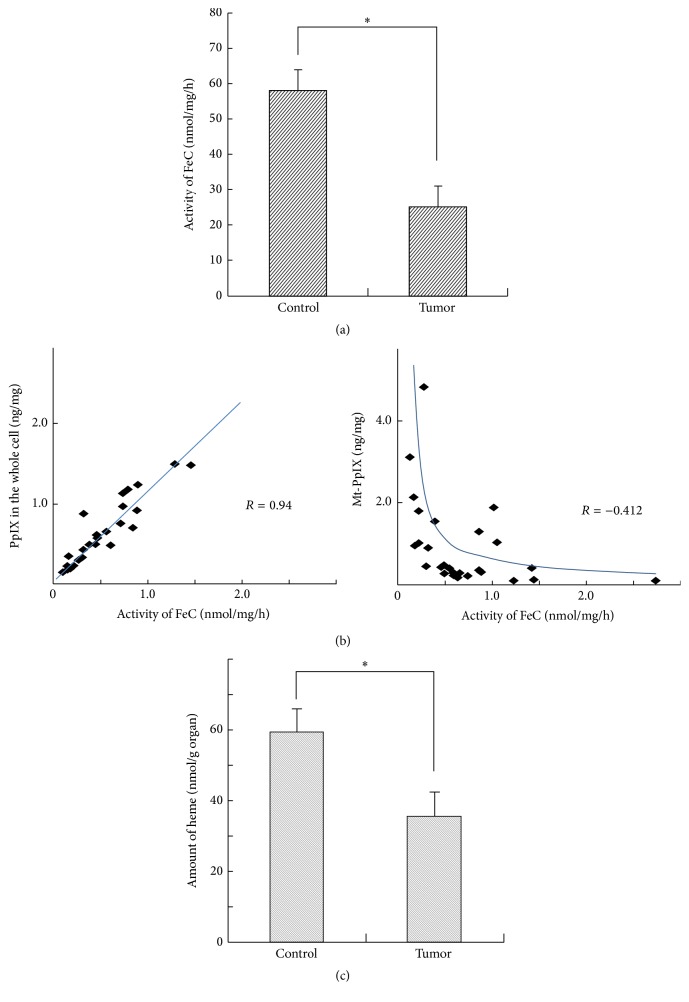
(a) Ferrochelatase (FeC) activity. Note that the FeC activity clearly decreased more in the malignant glioma cells (*n* = 28) than in the control cells (*n* = 14) (^*∗*^ *p* < 0.01). The FeC activity is expressed by the amount of heme biosynthesized from PpIX per unit time. Namely, the unit of FeC activity is nmol of heme formed/mg/organ/h. The unit of PpIX is nmol/g/organ. (b) The correlation between the FeC activity and the amount of PpIX in the mitochondria or in the whole cell. The left panel shows the relationship between the total intracellular amount of PpIX and the FeC activity. The right panel shows the relationship between the amount of PpIX in the mitochondria and the FeC activity. Note that the FeC activity and the total amount of PpIX were positively correlated (*R* = 0.94). The FeC activity and the amount of PpIX in the mitochondria were negatively correlated (*R* = −0.412). Namely, the FeC activity decreased with an increase in the accumulated amount of PpIX in the mitochondria. (c) The amount of heme in the mitochondria. The amount of heme in the mitochondria was compared between the control cells (*n* = 14) and tumor cells (*n* = 28). This amount was remarkably smaller in the tumor tissue than in the normal tissue (^*∗*^ *p* < 0.01).

**Figure 4:**
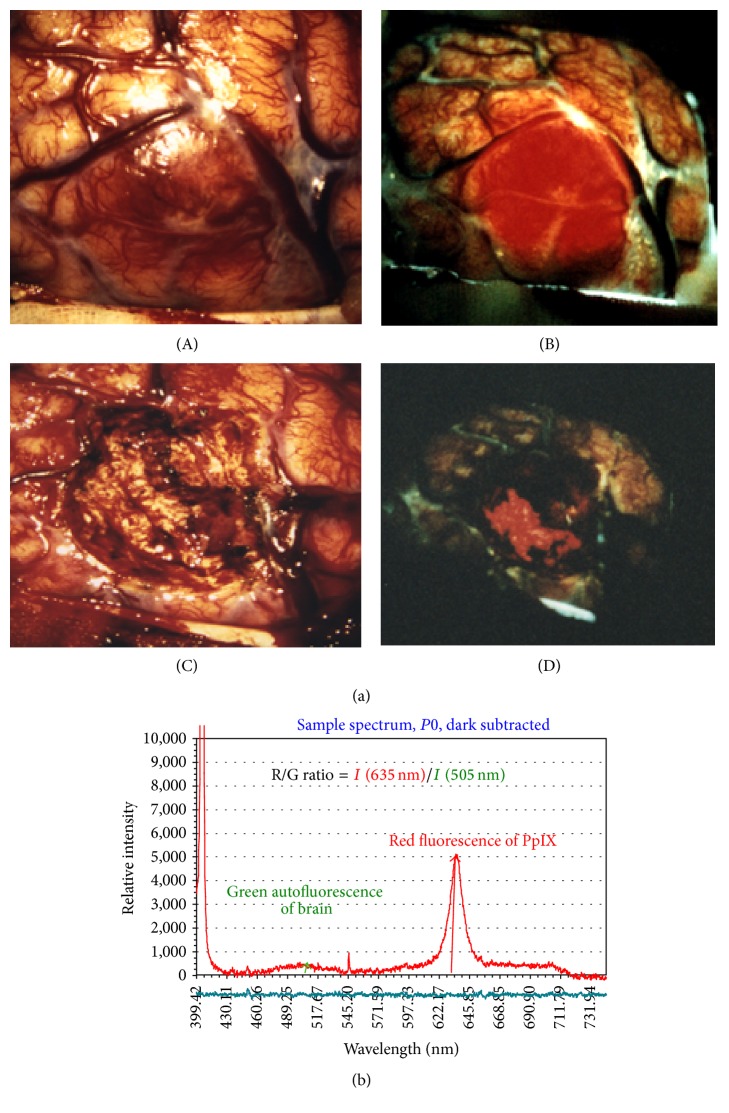
(a) An intraoperative photograph of the brain surface. (A) The tumor surface under xenon white light. Hypervascularity is observed; however, the tumor boundary is vague. (B) By irradiating excitation light of violet-blue, red fluorescence from the tumor tissues is observed and the tumor boundary is clearly recognized. (C) In the cavity after tumor removal under xenon white light, the residual tumor is not clearly recognized. (D) In the same operative field as (C) under excitation light of violet-blue, the residual tumor is clearly recognized. (b) The R/G ratio with fluorescence spectrum. We obtained the amplitude of the red (R) PpIX fluorescence from the glioma tissues and the green (G) autofluorescence from the glioma tissues in a spectrum.

After ALA administration, ALA dehydratase obviously increased and accelerated porphyrin metabolism from ALA along with ALA incorporation into malignant glioma cells. It is also obvious that the material that was excessively produced and accumulated was PpIX. To examine the mechanism underlying the increase in the amount of PpIX, the activities of three enzymes, ALA dehydratase, porphobilinogen deaminase (PBGD), and FeC, which are involved in porphyrin metabolism were simultaneously measured. In the malignant glioma cells, a decreasing trend in these enzymes with increasing amounts of porphyrin was observed. That is, the amount of PpIX and the activity of FeC were negatively correlated. The FeC activity was expressed as the amount of heme biosynthesized for PpIX per unit time. The unit of FeC activity is nmol of heme formed/mg/organ/h. The FeC activity was clearly decreased more in the malignant glioma cells compared with control cells (Figure 3(a)). The FeC activity and the total amount of PpIX in the whole cell were positively correlated; however, the FeC activity and the amount of PpIX in the mitochondria were negatively correlated (Figure 3(b)). We showed that, in the mitochondria, the FeC activity decreased with an increase in the accumulated amount of PpIX. These results indicated that the excessive presence of PpIX in the mitochondria inhibits the FeC activity in the malignant glioma cells. The amounts of Fe in the malignant glioma and the controls were 65.78 *μ*g/g and 92.89 *μ*g/g of wet brain, respectively. Although this was not statistically significant, the amount of Fe in the malignant glioma was obviously smaller than in the controls. The amount of heme in the mitochondria was smaller in the malignant glioma cells than in the control cells. This was thought to be because heme was not biosynthesized from PpIX, although a large amount of PpIX was present, due to a small amount of Fe and low FeC activity in the malignant glioma cells (Figure 3(c)). Based on these results, the reason why PpIX is selectively accumulated in malignant glioma cells can be explained on the basis of porphyrin-heme metabolism as follows. A large amount of PpIX is first biosynthesized in the mitochondria, and the excessive amount of PpIX in the mitochondria decreases the activity of FeC. Because the amount of Fe is small, the biosynthesis from PpIX to heme decreases. Consequently, the amount of heme in the mitochondria is smaller in the malignant glioma cell than in the control cell. We also speculated that FeC activity is decreased due to the activation of PBGD and the existence of nitric monoxide.

The other explanation as to why ALA or PpIX is oncotropic is speculated to be as follows. Since porphyrins have a high affinity to lipoprotein, especially to low-density lipoprotein, which is abundant in tumor cells, PpIX, which is a porphyrin, accumulates in the tumor cells by binding to low-density lipoproteins. However, there is no evidence that PpIX has a high affinity to low-density lipoprotein.

The pept-1 and pept-2 transporter controls cellular ALA uptake. In addition, the ATP-binding cassette transporter ABCG2 plays a critical role in regulating the cellular accumulation of porphyrin.

#### 3.1.4. Intracellular Localization of PpIX

We confirmed that PpIX fluorescence occurred in tissues with high cell density and in the cytoplasm of tumor cells by comparing the findings of HE stained, isolated preparations and confocal laser fluorescence microscopy [8].

### 3.2. Practical Aspects of ALA-PDD

#### 3.2.1. Diagnostic Accuracy

On histopathology, the very strongly fluorescent tissues were active tumor cells, and the weakly fluorescent tissues showed an infiltrative area at the tumor margin. The blue fluorescent tissues were normal brain tissue. Most necrotic lesions showed either very weak or no fluorescence.

Excessive accumulation occurs not only in malignant glioma tissues but also in other tissues. For example, inflammatory tissues in acne and granulation tissue accumulate PpIX because PpIX accumulation is the result of an abnormality in porphyrin metabolism.

The major drawbacks of PDD are the occurrence of false positive and false negative results. A false positive result is defined as the presence of fluorescence in the absence of histological confirmation of malignant glioma tissues. False positivity reflects the specificity of PDD. Conversely, a false negative result is defined as the absence of fluorescence despite the histological confirmation of malignant glioma tissues. False negativity reflects the sensitivity of PDD. Tissues yielding false positive results are often inflammatory cell infiltrates, and thus extirpation of the glioma, especially recurrent malignant glioma, requires careful consideration.

In our results, the specificity was 92.3%, and the sensitivity was 86.0% (Table 1).

False positivity is generally due to an abnormality in heme metabolism after PpIX synthesis from ALA. Therefore, false positive results are not confined to tumor tissues. If a similar abnormality is present in inflammatory tissues, PpIX accumulates in these tissues and fluorescence is observed.

#### 3.2.2. Photobleaching during Surgery

If photobleaching of porphyrins by illumination of the microscope occurs immediately, this is a very important issue for the diagnosis of malignant glioma during surgery. Stummer et al. [5, 9, 10] reported that, under illumination of the microscope, the fluorescence decreased to 36% in 25 min for violet-blue light and in 87 min for white light excitation. In addition, they reported that photobleaching was much slower than had been anticipated. Even if fluorescence decreases in the exposed regions of the tumor, it can be refreshed by immediate removal of the superficial tissue. Therefore, we believe that photobleaching is not such a serious issue during surgery.

#### 3.2.3. The Relationship between the Malignancy and the Amount of PpIX in the Tissue

**Figure 5:**
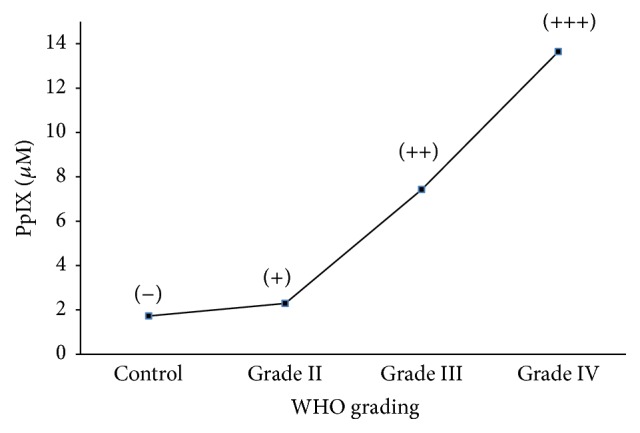
The amount of PpIX accumulation in each grade of glioma. High-grade malignant glioma contains a high concentration of PpIX in the tissue. This sign of (−)~(+++) shows the intensity of fluorescence to the naked eye.

We measured the amount of PpIX in each grade of glioma. While the amount in control brain tissue was 1.73 *μ*M (micromole), it was 2.29 *μ*M in WHO grade II glioma, 7.43 *μ*M in WHO grade III anaplastic astrocytoma, and 13.65 *μ*M in WHO grade IV glioblastoma (Figure 5). The amount of PpIX was 1.8 times greater in grade II, 4.3 times greater in grade III, and 7.9 times greater in grade IV compared with the concentration in normal brain tissue. The amount of intracellular PpIX increased exponentially with the increase in malignancy.

#### 3.2.4. The Fluorescence Spectrum with ALA-PDD Is Most Useful for the Estimation of Tumor Size during Surgery

We examined the tumor size that could be recognized by histopathological findings, the R/G ratio in the fluorescence spectrum, the visual fluorescence strength, and the visual observation under the white light.

**Figure 6:**
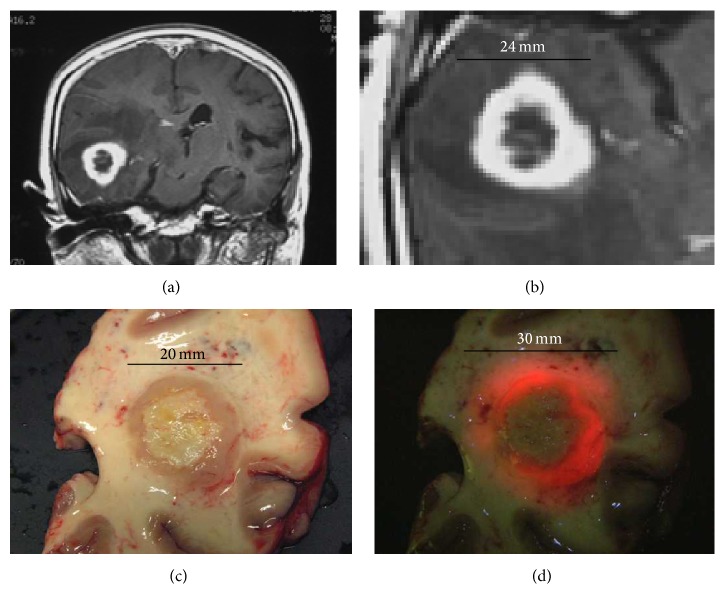
The size of the tumor using different methods. (a) MRI image shows the glioblastoma in the right temporal lobe. (b) It was 24 mm on enhanced MRI. (c) It was 20 mm on macroscopic observation under the white light. (d) It was 30 mm on macroscopic observation and fluorescence to the naked eye.

This case was a female with right temporal lobe glioblastoma. An MR image with gadolinium contrast is shown in Figure 6(a) and a tumor shadow is observed in the right temporal lobe. Figures 6(c) and 6(d) show the cut surface of the central portion of the tumor after total resection of the right temporal lobe glioblastoma.  Figure 6(c) is a picture of the surface under white light and the tumor is recognized by its brownish color.  Figure 6(d) is the same surface as Figure 6(c) under excitation violet-blue light and the fluorescence of the tumor is clearly recognized.

**Figure 7:**
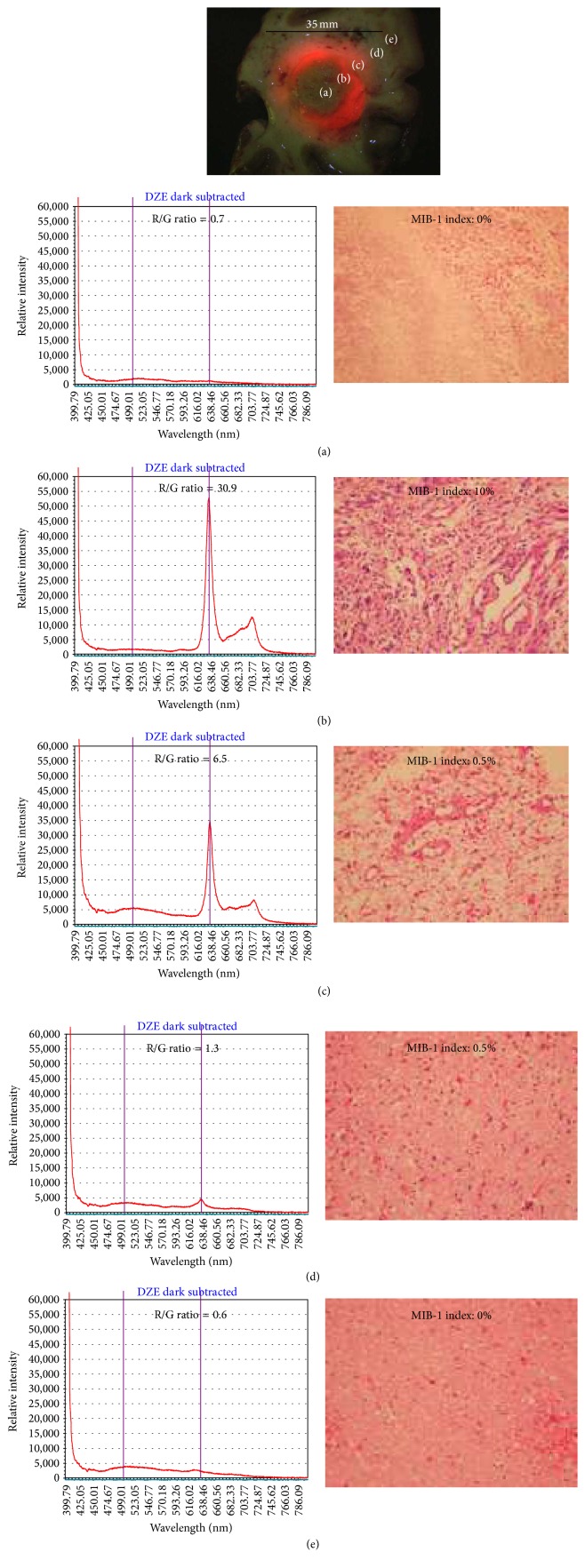
The analysis of the fluorescence spectrum and the histology in each lesion. Lesion (a) was central necrosis without macroscopic fluorescence. Lesion (b) showed the highest fluorescence macroscopically. Lesion (c) showed a faint fluorescence macroscopically. Lesion (d), adjacent to lesion (c), did not show macroscopic fluorescence. Lesion (e) did not show any fluorescence. The left panel shows the analysis of the fluorescence spectrum, and the right panel shows the histological examinations and MIB-1 index.

Whether the tumor could be recognized or not was examined in five lesions (lesions (a), (b), (c), (d), and (e)) as shown in Figure 7. Apparent fluorescence was not observed macroscopically in lesion (a) (Figure 7(a)). Histopathologically, lesion (a) was composed of necrotic tissue and did not show the band at 635 nm that represents PpIX. Apparent strong fluorescence was observed macroscopically in lesion (b) (Figure 7(b)). Histopathologically, this lesion was active glioblastoma with abnormal nuclei and a high cell density, and its MIB-1 index was 10%. In lesion (b), a very strong peak at 635 nm was observed, and the ratio of autofluorescence to PpIX fluorescence (R/G ratio) was 30.9. In lesion (c) (Figure 7(c)), weak fluorescence was recognized macroscopically and histopathologically, and most of the tumor was identified as a low-grade glioma infiltrating into the normal brain tissue. The MIB-1 index of lesion (c) was 0.5%, and its spectrum showed a relatively strong peak at 635 nm, and the R/G ratio was 6.5. In lesion (d) (Figure 7(d)), no fluorescence was detected macroscopically with the naked eye. However, its spectrum showed a very weak peak at 635 nm and the R/G ratio was 1.3. The tissue in lesion (d) was identified histopathologically as normal brain tissue with a few infiltrating glioma cells (guerilla cells). Its MIB-1 index was 0.5%. In lesion (e) (Figure 7(e)), no fluorescence was observed macroscopically, and no peak was observed at 630 nm. The tissue in lesion (e) was confirmed on histopathology to be normal brain tissue. The greatest diameter of the glioma that was distinguished from the normal tissue was 35 mm (Figure 7). In this range, the spectrum showed an obvious band at 635 nm, and it was histopathologically confirmed as glioma tissue.

These results suggest that fluorescence diagnosis during surgery can successfully distinguish malignant gliomas of a size of 35 mm from normal tissue. Furthermore, the fluorescence spectrum during surgery can be used for diagnosis, even if fluorescence is not observed macroscopically. As a method for diagnosing a malignant glioma from the normal brain during surgery, we can say that the fluorescence spectrum has excellent diagnostic capability.

#### 3.2.5. Curative Effects of ALA-PDD

We obtained the following surgical results using ALA-PDD. The total removal rate of the glioblastoma before using ALA-PDD was 40%. However, the total removal rate after using ALA-PDD rose to 78%.

## 4. Discussion

### 4.1. The Value of ALA-PDD for Malignant Glioma Surgery

Tumor resection using fluorescence of ALA-induced PpIX does not differ greatly from conventional neurosurgical microsurgery.

Many methods are used to distinguish the border of the malignant glioma from the normal brain. For example, there are neuronavigation systems, ultrasonography, or MRI during surgery. Tumor resection using fluorescence of ALA has now been added to these methods and probably represents the best one. Although these methods can distinguish the border of the malignant glioma pathologically, unfortunately, these methods cannot distinguish the regions of brain function. When malignant glioma cells infiltrate into the eloquent brain area that is a center of speech or motor function with fluorescence, speech and motor function must be monitored to avoid resection of the eloquent areas with malignant glioma [11]. Therefore, PDD using ALA can distinguish malignant glioma more widely than MRI. Distinguishing malignant glioma using fluorescence has superior specificity compared with neuronavigation systems, ultrasonography, or MRI [12].

Stummer et al. [5, 10, 13] described the clinical curative effects of ALA-PDD as follows. The total removal rate by resection surgery of the glioblastoma before using ALA-PDD was 36%. However, the total removal rate after using ALA-PDD rose to 66%. In cases of total resection of fluorescing tissues, the mean survival time was 101 weeks (23.5 months). In cases where solid fluorescing tissues were left unresected, the mean survival time was 51 weeks (11.9 months). In addition, 6-month progression-free survival was 40.1%–46.0% in the GFR group and 21.1%–28.3% in the controls.

## 5. Conclusions

Photodynamic application using ALA is a new and promising technique for neurosurgeons in the treatment of malignant glioma. ALA-PDD has succeeded in increasing the resection rate of malignant glioma from 40% to 80%. PDD significantly prolongs the interval until recurrence. Furthermore, with intraoperative PDD for identifying tumor tissues, measurement of the fluorescent spectrum facilitates more accurate and extensive resection compared with macroscopic observation of the fluorescence.

Based on previous research, it is apparent that PpIX accumulates abundantly in malignant glioma tissues after ALA administration. Furthermore, it is evident that the mechanism underlying PpIX accumulation in malignant glioma tissues involves an abnormality in porphyrin-heme metabolism, specifically decreased FeC enzyme activity.

Although chemotherapy with new anticancer agents, immunotherapy, and new methods of radiotherapy and gene therapy will be developed in the future, we believe that the curative effect of this new treatment has the potential to show a maximum effect by maximum cytoreduction with photodynamic application using ALA. Therefore, we strongly believe that photodynamic application using ALA will play a key role in malignant glioma treatment before the development of new treatments in the future.

Many ALA derivatives have been synthesized by ALA research. For example, there are ALA ether, methyl-ALA, and hexyl-ALA. These new derivatives have been developed because it is known that their tumor affinity is higher than ALA. Furthermore, these new derivatives may be more suitable for fluorescence detection because they appear to induce PpIX with better tumor selectivity. Our hope is that these new ALA derivatives will be used in the future.

Furthermore, ALA-PDT (photodynamic therapy) has the potential to treat malignant gliomas near or at the eloquent area of the brain without major neurological deficits [14]. It is known that photodynamic application using ALA is also effective for brain tumors other than malignant glioma, including pituitary adenomas, metastatic brain tumors, and malignant meningiomas that have invaded into the skull base.

From our clinical experience, the following advantages and disadvantages are apparent. Advantages are as follows: (1) This method was able to distinguish malignant brain tumor tissues from normal brain tissues with the naked eye before tumor removal at the time of removal surgery. (2) Photodynamic medicine has few side effects and provides a good quality of life. Disadvantages are as follows: (1) Using PDD, it is very difficult to distinguish infiltrative tumor tissues or guerilla cells from normal tissues. Furthermore, there are false negative and false positive findings especially in edematous brain tissues or recurrent tumor tissues. (2) The concentration of the photosensitizer in malignant brain tumor tissues is heterogeneous.

## Figures and Tables

**Table 1:** The relationship between the fluorescence and the pathological examination.

*n* = 217	Glioma cells	Nonglioma cells
Fluorescence (+)	153	3 (all of them were necrosis tissues)
Fluorescence (−)	25	36

Sensitivity: 86.0%; specificity: 92.3%.

## References

[1] Committee of Brain Tumor Registry of Japan (2014). Report of brain tumor registry of Japan (2001–2004). *Neurologia Medico-Chirurgica*.

[2] Lacroix M., Abi-Said D., Fourney D. R. (2001). A multivariate analysis of 416 patients with glioblastoma multiforme: prognosis, extent of resection, and survival. *Journal of Neurosurgery*.

[3] Keles G. E., Anderson B., Berger M. S. (1999). The effect of extent of resection on time to tumor progression and survival in patients with glioblastoma multiforme of the cerebral hemisphere. *Surgical Neurology*.

[4] Kaneko S. (2001). Intraoperative photodynamic diagnosis of human glioma using ALA induced protoporphyrin IX. *Neurological Surgery*.

[5] Stummer W., Pichlmeier U., Meinel T., Wiestler O. D., Zanella F., Reulen H.-J. (2006). Fluorescence-guided surgery with 5-aminolevulinic acid for resection of malignant glioma: a randomised controlled multicentre phase III trial. *The Lancet Oncology*.

[6] Kondo M. (1995). Methods of determination of porphyrins and their precursors—introduction of analytical methods for porphyrin metabolites. *Nippon Rinsho*.

[7] Harada K., Ohmori S., Kim Y., Tomokuni K. (1995). Metabolic fate of porphyrin and its precursors in porphyria and porphyrinuria. *Nippon Rinsho*.

[8] Fukuda H., Paredes S., Battle A. M. C. (1992). Tumour-localizing properties of porphyrins *in vivo* studies using free and liposome encapsulated aminolevulinic acid. *Comparative Biochemistry and Physiology Part B: Comparative Biochemistry*.

[9] Stummer W., Stocker S., Novotny A. (1998). In vitro and in vivo porphyrin accumulation by C6 glioma cells after exposure to 5-aminolevulinic acid. *Journal of Photochemistry and Photobiology B: Biology*.

[10] Stummer W., Novotny A., Stepp H., Goetz C., Bise K., Reulen H. J. (2000). Fluorescence-guided resection of glioblastoma multiforme by using 5-aminolevulinic acid-induced porphyrins: a prospective study in 52 consecutive patients. *Journal of Neurosurgery*.

[11] Kaneko S. (2008). A current overview: photodynamic diagnosis and photodynamic therapy using 5-aminolevulinic acid in neurosurgery. *Nippon Laser Igakkaishi*.

[12] Kaneko S. (2011). Photodynamic therapy for human malignant glioma. *Nippon Laser Igakkaishi*.

[13] Stummer W., Tonn J.-C., Mehdorn H. M. (2011). Counterbalancing risks and gains from extended resections in malignant glioma surgery: a supplemental analysis from the randomized 5-aminolevulinic acid glioma resection study: clinical article. *Journal of Neurosurgery*.

[14] Kaneko S., Okura I., Tanaka T. (2015). Photodynamic applications (PDD, PDT) using aminolevulinic acid in neurosurgery. *Aminolevulinic Acid*.

